# Electroretinogram Monitoring of Dose-Dependent Toxicity after Ophthalmic Artery Chemosurgery in Retinoblastoma Eyes: Six Year Review

**DOI:** 10.1371/journal.pone.0084247

**Published:** 2014-01-20

**Authors:** Jasmine H. Francis, David H. Abramson, Y. Pierre Gobin, Brian P. Marr, Ira J. Dunkel, Elyn R. Riedel, Scott E. Brodie

**Affiliations:** 1 Ophthalmic Oncology Service, Memorial Sloan-Kettering Cancer Center, New York, New York, United States of America; 2 Department of Ophthalmology, Weill Cornell Medical College, New York, New York, United States of America; 3 Service of Interventional Neuroradiology, Departments of Neurosurgery Neurology and Radiology, Weill Cornell Medical College of New York Presbyterian Hospital, New York, New York, United States of America; 4 Department of Pediatrics, Memorial Sloan-Kettering Cancer Center, New York, New York, United States of America; 5 Department of Pediatrics, Weill Cornell Medical College, New York, New York, United States of America; 6 Department of Epidemiology and Biostatistics, Memorial Sloan-Kettering Cancer Center, New York, New York, United States of America; 7 Department of Ophthalmology, Mount Sinai School of Medicine, New York, New York, United States of America; Institute of Clinical Physiology, Italy

## Abstract

**Purpose:**

To report electroretinogram responses of retinoblastoma children under anesthesia before and after treatment with chemotherapeutic drugs (melphalan, topotecan, carboplatin) delivery by ophthalmic artery chemosurgery (OAC).

**Methods:**

A cohort study of 81 patients with retinoblastoma treated with OAC. All patients treated with OAC at our center through May 2012 for whom the requisite ERG data were available are included in the analysis. This study recorded the ERG 30 Hz flicker amplitude response changes from baseline, at 3 and 12 months following OAC treatment completion. Both univariate and multivariate linear regression models were evaluated, with generalized estimating equations to correct for correlations within patients. Independent numerical variables included maximum doses and cumulative doses of melphalan, topotecan and carboplatin.

**Results:**

By univariate analysis, both melphalan and topotecan appear to be associated with changes in ERG amplitude at both 3 and 12 months; but for the most part, these changes are minimal and likely clinically insignificant. By multivariate analysis, maximum and cumulative melphalan have a modest, temporary effect on the ERG amplitude change, which is apparent at 3 months but no longer evident at 12 months after completing treatment. By multivariate analysis, topotecan and carboplatin do not appear to adversely effect the change in ERG response.

**Conclusion:**

Melphalan has the strongest, and carboplatin the weakest association with reduction in ERG response amplitudes; but for the most part, these changes are minimal and likely clinically insignificant. These conclusions apply only over the dose ranges used here, and should be applied with caution.

## Introduction

Seven years ago, we first started treating retinoblastoma with ophthalmic artery chemosurgery (OAC), with the initial intent to save eyes with extensive retinoblastoma destined for enucleation. This treatment consists of introducing a micro-catheter into the femoral artery and advancing it to the orifice of the ophthalmic artery where chemotherapy is injected. It allows for concentrated, localized administration of chemotherapy to the eye while minimizing systemic exposure and limiting systemic toxicity. The elevated local dose– reported to be three-fold the plasma dose in non-tumor bearing animals receiving OAC with melphalan [1], and 10-fold the dose compared to periocularly administered topotecan [2] (and probably higher in diseased retinoblastoma eyes)– raises concern for ocular toxicity since prior animal studies have demonstrated dose-related retinal toxicity.

With OAC, the chemotherapy disperses along the territory of the ophthalmic artery and could conceivably produce toxic effects anywhere along this distribution. We have previously reported on reversible cutaneous hyperemia following OAC in the vascular distribution of the supratrochlear artery (a distributary extending from the ophthalmic artery) [3]. The ophthalmic artery also gives rise to the central retina artery, delivering drug directly to the inner retina and accordingly, our protocol calls for electroretinography as a measure of retinal toxicity. We have previously reported on the ERG findings of our first 10 patients, demonstrating that ERG responses can persist and even recover at 3–14 months following OAC with mostly one, but also two drugs [4]. And we have briefly summarized our ERG findings, including eyes with >50% retinal detachment [5]–[7].

Now that treatment advancements for retinoblastoma have allowed for improved patient and ocular survival, the prospect of saving vision draws more attention to retinal toxicity. For example, in the treatment of the subset of advanced Reese-Ellsworth group V eyes with >50% retinal detachment, OAC has surpassed all historical experience with treatment success rates almost twice those obtained with intravenous chemotherapy [5]. With advanced eyes being saved and with an impressive rate of retinal reattachment in these eyes (76%), along with the utility of OAC being extended to less advanced eyes, there is now a larger pool of treated eyes with visual potential. This highlights the importance of understanding the impact of OAC on retinal function. We have chosen to address this through ERG measurements, particularly in these preverbal patients in whom Snellen visual assessments cannot be obtained.

This report is a comprehensive analysis of our data, for eyes treated with one, two or three drugs (melphalan, topotecan and carboplatin) since our protocol started 6 years ago. In the more than 20 peer-reviewed publications on OAC thus far, there are none that document ERG findings on a group of OAC-treated eyes as large as this.

## Materials and Methods

### Ethics Statement

Written informed consent was obtained from the parents, caretakers or guardians on behalf of all children and placed into the patient record. Parents/caretakers/guardians gave consent for this established, published treatment for an off-label use of chemotherapy. The Memorial Sloan-Kettering Institutional Review Board approved this study and provided an exemption for the review of these patients. Patients would have received these treatments regardless of their inclusion in this study. All authors, who represented the team of physicians caring for these patients, collectively made the treatment decision.

This study included all eyes treated by OAC at Memorial Sloan-Kettering between May 2006 (when we first began OAC treatment) and May 2012 for whom the requisite ERG data were available. Inclusion criteria consisted of retinoblastoma eyes treated with OAC with ERG measurements a. before OAC, b. 3 months after the last OAC treatment, +/−1 month and, if available, c. 12 months after last OAC treatment, +/−2 months.

The entire cohort of eyes treated by OAC between May 2006 and May 2012 comprises 161 eyes. Of these, ERG measurements at baseline were available for 139 eyes. ERG measurements at three-month follow-up after the final OAC treatment were available for 103 of these eyes in 81 patients, which form the basis for the present study. ERG data at the 12-month follow-up was available for 64 eyes.

ERG data were unavailable only for incidental reasons unrelated to the clinical status of the patients, such as malfunction of the ERG recording instrument, unavailability of an electrophysiologist to perform the ERG study during the EUA, or failure of the patient to keep a scheduled appointment. Apart from absence of the ERG data, there were no other exclusion criteria for this study.

The intra-arterial technique has been previously described in detail [8].

### Electroretinography

ERG recordings were obtained during regularly scheduled examination under anesthesia, according to an ISCEV standard protocol which had been modified to limit anesthesia time, as previously described [9]. Reported here are the response amplitudes to 30-Hz photopic flicker stimulation, which are representative of the full protocol. In brief, ERGs were obtained using a hand-held ganzfeld stimulator (Espion ColorBurst, Diagnosys LLC, Lowell, MA) and ERG-jet contact lens electrode. Light adapted 3.0 single flash and 30 Hz flicker responses were obtained singly, and then averaged in groups of 10, with the averaged waveforms employed for analysis.

The response to 30-Hz flicker stimulation was used to represent the complete set of ERG responses, since photopic and scotopic responses were highly correlated, and the 30-Hz stimulus enabled signal detection in severely impaired retinas [10]. Furthermore, the 30 Hz flicker response has the advantage of a shortened protocol affording reduced anesthesia time and a repetitive waveform that facilitates further discrimination between signal and noise. While the 30 Hz flicker has the disadvantage of not distinguishing the selective photoreceptor effects of a possible paraneoplastic process, this has not been observed in these retinoblastoma eyes. A change in 30-Hz response amplitude of 25 µV was considered clinically meaningful, based on statistical analysis of ERGs during EUA of normal eyes (unpublished data).

### Statistical Analysis

Associations were evaluated between treatment (maximum doses and cumulative doses of melphalan, topotecan and carboplatin) and the change from baseline in 30 Hz flicker response amplitude at both 3 months and 12 months after OAC completion, before and after adjusting for Reese-Ellsworth (RE) Classification and prior treatment (defined as previous radiation or systemic chemotherapy). Linear regression models were used with generalized estimating equations to correct for correlations within patients. Amplitude variables consisted of 1. Change from baseline, 2. Change from baseline with values capped at 100 µV, 3. Change from baseline standardized by weight, 4. Change in baseline with patients removed who had alterations in retinal architecture (ie. resolution or development of retinal detachment). It is common for drugs, particularly chemotherapy, to be dosed based on weight or body surface area to improved efficacy and limit toxicity. For this reason, the authors thought it important to understand the drug toxicity of ophthalmic artery chemotherapy based on patient weight. In addition, we have observed that general anesthesia frequently produces supra-normal, but highly variable electroretinogram responses (as compared with normative data from awake patients – report in preparation). Since flicker responses in awake patients rarely exceed 100 µV in amplitude the authors thought it worthwhile to repeat the data analysis with supra-normal responses truncated to a value of 100 µV, thereby mitigating any influence from data fluctuations in the supra-normal range. (We are reassured that this replicate analysis reached similar conclusions to the primary analysis of the original data).

A potential cutpoint was estimated for the effect of maximum and cumulative drug dose on change in amplitude from baseline to 3 months post treatment using a maximally selected Wilcoxon rank statistic. Statistical computations were performed using SAS (version 9.2, SAS Institute, Cary, NC, USA) and Maxstat package (version 0.7–9) in R (version 2.3.1, R Foundation for Statistical Computing, Vienna, Austria).

## Results

103 eyes in 81 patients were included in this study. All 103 eyes had ERG data 3 months following last OAC, and of these, 64 eyes had ERG data 12 months following last OAC. The mean and median patient weight was 11.3 Kg and 10 Kg, respectively. 44 eyes (43%) had received prior treatment while 59 eyes (57%) were naïve to treatment. 79 eyes (77%) were RE Group V, while the remaining eyes were classified as RE Group I–IV. Neither RE classification (p = 0.31) nor prior treatment status (p = 0.16) was statistically associated with changes in ERG amplitude. The mean and median drug doses are shown Table 1, and the mean and median 30 Hz Flicker responses are indicated in Table 2. Univariate and multivariate analyses of ERG response changes are shown in Tables 3 and 4, respectively. Changes in the other amplitude variables (capped at 100 µV, standardized by weight, patients removed who had alterations in retinal architecture) yielded similar results to those shown in Tables 3 and 4.

**Table 1:** Mean, median and range of maximum and cumulative drug doses used during ophthalmic artery chemosurgery in 103 eyes.

Drug	Variable	Mean (std)	Median (IQR)	Range
Melphalan	Max dose (mg)	4 (1.7)	4 (3–5)	0–7.5
	Cum dose (mg)	12.9 (9.4)	9 (7–16.5)	0–45
Topotecan	Max dose (mg)	0.3 (0.2)	0.3 (0–0.4)	0–1
	Cum dose (mg)	0.9 (0.8)	0.7 (0–1.4)	0–3.3
Carboplatin	Max dose (mg)	18.3 (18.5)	25 (0–30)	0–60
	Cum dose (mg)	42 (58.2)	25 (0–60)	0–300

std = standard deviation, IQR = interquartile range.

**Table 2:** Mean, median and range of electroretinogram (ERG) response at baseline, 3 mos and 12 mos and change in ERG at 3 mos and 12 mos following ophthalmic artery chemosurgery.

	Amplitude variable	N	Mean (std)	Median (IQR)	Range
	Baseline	103	66.4 (51.8)	66.7 (17.6 to 103.3)	0 to 198.6
30 Hz Flicker response (µV)	3 mo	103	66 (51.9)	55.4 (24.5 to 97.7)	0 to 229
	12 mo	64	70.3 (56.4)	63.1 (26.2 to 103.1)	0 to 257
	All (µV)	103	−0.4 (39.2)	−0.2 (−20.2 to 24.9)	−112.6 to 120.8
Change BL to 3 mos	Values capped at 100 µV (µV)	103	−0.3 (27.6)	0 (−9.4 to 11.6)	−100 to 78.6
	Standardized by weight (µV/Kg)	103	0.2 (4.1)	0 (−1.9 to 2.4)	−12.4 to 14.6
	All (µV)	64	8.4 (46.4)	2.2 (−18.6 to 37.4)	−102.9 to 136.9
Change BL to 12 mos	Values capped at 100 µV (µV)	64	5.1 (30.6)	0.2 (−5 to 23.5)	−97.3 to 87.9
	Standardized by weight (µV/Kg)	64	1.2 (5)	0.2 (−1.6 to 3.9)	−10.1 to 16.5

std = standard deviation, IQR = interquartile range.

**Table 3:** Univariate regression analysis of electroretinogram response changes using linear regression models.

Chemo Variable	Amplitude Variable	Estimate (µV)	GEE p-value	Adjusted Estimate* (µV)	GEE Adjusted p-value*
Maximum Melphalan	Change BL to 3 mos	−6.7	0.005	−6.2	0.015
	Change BL to 12 mos	−10.4	0.018	−10.1	0.036
Cumulative Melphalan	Change BL to 3 mos	−1.5	0.003	−1.4	0.008
	Change BL to 12 mos	−1.6	0.021	−1.5	0.041
Maximum Topotecan	Change BL to 3 mos	−46.2	0.017	−42.8	0.037
	Change BL to 12 mos	−70.3	0.014	−70.3	0.014
Cumulative Topotecan	Change BL to 3 mos	−12.2	0.022	−11.1	0.062
	Change BL to 12 mos	−18.6	0.016	−18.1	0.027
Maximum Carboplatin	Change BL to 3 mos	−0.4	0.072	−0.4	0.101
	Change BL to 12 mos	−0.8	0.02	−0.8	0.021
Cumulative Carboplatin	Change BL to 3 mos	−0.1	0.188	−0.1	0.272
	Change BL to 12 mos	−0.2	0.099	−0.2	0.089

GEE = generalized estimating equation, BL = baseline (before OAC),

* = These results are based on multivariate models that adjust for Reese-Ellsworth Classification and prior treatment.

**Table 4:** Multivariate regression analysis of electroretinogram response changes using linear regression models.

Amplitude Variable	Chemo Variable	Estimate (µV)	GEE p-value	Adjusted Estimate*(µV)	GEE Adjusted p-value*
	Maximum Melphalan	−4.71	**0.04**	−4.32	0.06
Change BL to 3 mos	Maximum Topotecan	−22.5	0.21	−21.21	0.24
	Maximum Carboplatin	−0.12	0.57	−0.13	0.56
	Maximum Melphalan	−5.23	0.19	−4.81	0.29
Change BL to 12 mos	Maximum Topotecan	−43.88	0.12	−43.81	0.11
	Maximum Carboplatin	−0.47	0.09	−0.48	0.08
	Cumulative Melphalan	−1.8	**0.01**	−1.76	0.01
Change BL to 3 mos	Cumulative Topotecan	3.41	0.64	3.78	0.62
	Cumulative Carboplatin	0.03	0.74	0.03	0.75
	Cumulative Melphalan	−0.58	0.53	−0.5	0.59
Change BL to 12 mos	Cumulative Topotecan	−13.58	0.23	−13.67	0.21
	Cumulative Carboplatin	0	0.98	0	0.98

GEE = generalized estimating equation, BL = baseline (before OAC),

* = These results are based on multivariate models that adjust for Reese-Ellsworth Classification and prior treatment.

**Figure 1:**
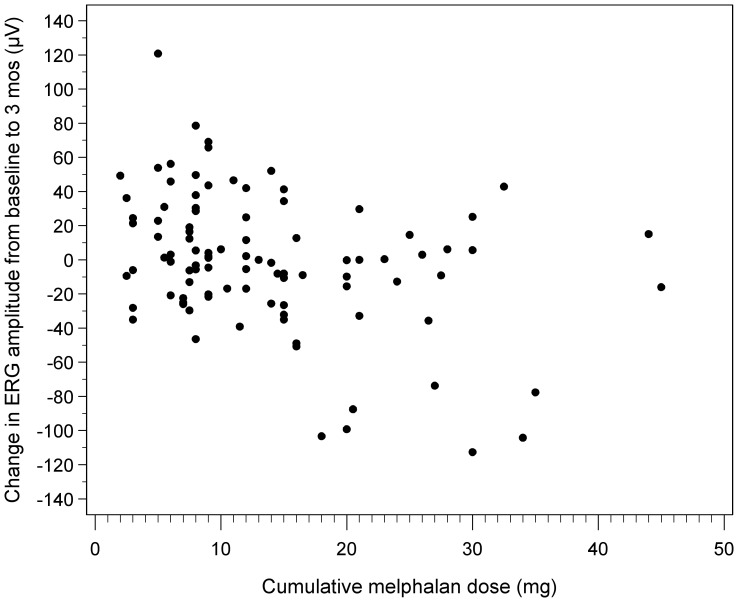
Scatterplot demonstrating change in ERG response to cumulative dose of melphalan over all data. Although a cutpoint of 14(p-value = 0.01), there remains variability above and below this dose.

**Figure 2:**
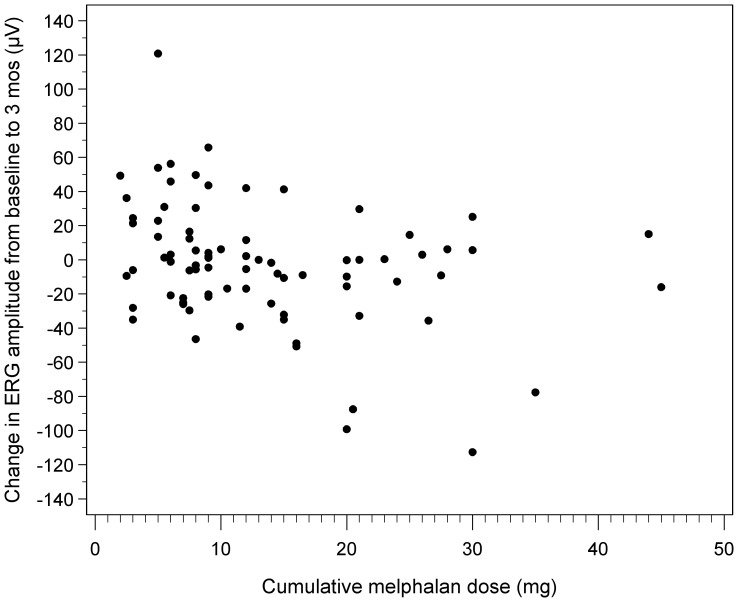
Scatterplot demonstrating change in ERG response to cumulative dose of melphalan after excluding patients with alterations in retinal architecture. A cutpoint of 10(p-value = 0.05); however, there is variability above and below this dose.

For cumulative melphalan dose and change in ERG amplitude from baseline to 3 months, there was a statistically significant estimated cutpoint at 14 mg (p-value = 0.01), and after excluding patients with alterations in retinal architecture, the estimated cut-point was calculated at 10 mg (p-value = 0.05). Scatterplots are shown in figures 1 and 2. The estimated cutpoint analysis was not significant for maximum dose of melphalan, topotecan nor carboplatin, nor for cumulative dose of topotecan or carboplatin.

### Melphalan

By univariate analysis, for every 1 mg increase in maximum melphalan dose, the ERG decreased by 6.7 µV at 3 months and by 10.4 µV at 12 months; and for every 1 mg increase in cumulative melphalan dose, the ERG decreased by 1.5 µV at 3 months and by 1.6 µV at 12 months (table 3). By multivariate analysis, melphalan had a statistically significant effect on the ERG amplitude change at 3 months: for every 1 mg increase in maximum or cumulative melphalan dose, the ERG decreased by 4.7 µV or 1.8 µV, respectively (table 4). This effect was no longer significant at 12 months (although this may be due to a smaller sample size).

### Topotecan

By univariate analysis, for every 0.1 mg increase in maximum topotecan dose, the ERG decreased by 4.6 µV at 3 months and by 7.0 µV at 12 months (table 3). For every 0.1 mg increase in cumulative topotecan dose, the ERG decreased by 1.2 µV at 3 months and by 1.9 µV at 12 months (table 3). By multivariate analysis, topotecan does not appear to affect the ERG response at either 3 or 12 months (table 4).

### Carboplatin

By univariate analysis, for every 10 mg increase in maximum carboplatin dose, the ERG change was not significantly effected at 3 months and decreased by 8.3 µV at 12 months (table 3). For every 10 mg increase in cumulative carboplatin dose, the ERG change was not significantly effected at either 3 or 12 months. By multivariate analysis, there was no significant effect on the ERG by carboplatin– however, this may be a result of smaller sample size.

## Discussion

There is no standard dosing formula that we employ for patients receiving OAC for retinoblastoma, since the dose selection is a dynamic process [8]. The choice of number of drugs used and dosage considers a combination of many factors including age and weight of patient, status of disease, blood flow and vascular territory; and may change for each eye at every OAC session. Relating any parameter to dose can be complex and as such, we use maximum and cumulative dose of each drug as a means of comparison between eyes.

The literature reports on a number of chorioretinal abnormalities associated with OAC infusions of chemotherapy, in both humans and a nonhuman primate model [11], [12]. Each clinical series consists of cohorts ranging from 12 to 408 patients [8], [13]–[21]. Reports of retinal pigment disturbances after OAC include a range of 5–53% of patients, retinal detachments in 15–27%, vitreous hemorrhage in 13–27%, ischemia or vasculopathies in 4–24% and choroidal disturbances in 0.5–29%. It may come as no surprise that a higher percentage of adverse events occurred in the smaller published cohorts (and likely represented the initial patients attempted with the OAC technique). The lowest adverse event rates are reported in the largest cohorts: 4% of 95 patients had an avascular retinopathy [8] and 0.5% of 408 patients exhibited “diffuse chorioretinal atrophy” [19]. It is unclear whether these reported changes are a result of drug toxicity, ophthalmic artery cannulation and/or competency of technique, wedge flow, prior or additional treatment, inter-observer variability, or tumor effects. Even if due to a drug effect, the variation in the number of drugs and in their dosing between these cohorts, makes it difficult to estimate meaningful information on individual drug toxicity.

It is important to recognize that, with the advent of OAC, the retinoblastoma battle has shifted to a very different spectrum of patients: we are saving eyes that would historically have been enucleated. It is these dismal eyes that are the subject of the present report on retinal toxicity. Given the inherently non-standardized methodology of OAC, which makes a standardized analysis of toxicity impossible, we have done our best to analyze the data in an informative way,

We fully recognize that the electroretinogram is only a proxy for subjective measurements of vision, employed out of necessity since most of our patients cannot verbalize their acuity, and visual evoked potential (VEP) is not appropriate during examination under anesthesia (since it suppresses the very same cortical neuron responses that one is trying to measure). Finally, note that as there is substantial variation in the baseline ERG responses due to tumor size, retinal detachment, previous treatment, we report primarily on *changes* of ERG amplitudes in order to account and to compensate for the baseline variation.

### Effect of Melphalan

Attempts have been made to understand the efficacy and toxicity of melphalan, topotecan or carboplatin given as a single dose. For instance, based on cell culture studies, 4 µg/ml melphalan is sufficient for retinoblastoma suppression [22]. Studies have attempted to further refine the upper limit of melphalan dosing using ERG as a measure of retinal toxicity. In rabbits, an intravitreal dose of 10 µg (corresponding to 5.9 µg/ml) was nontoxic to the retina by ERG [23], [24]. Initially, *in vivo* studies in rabbits and humans determined up to 40 µg/ml to be non-toxic by ERG [23], but intravitreal dosing in rabbits of 20 µg (approximately 11.8 µg/ml) were shown to “moderately” change the ERG. The ERG was seen to “greatly deteriorate” with a dose of 90 µg [24]. And in rabbits receiving melphalan infusion during vitrectomy, melphalan doses of 5 µg/ml had no adverse effect on ERG amplitude [25]. However perfusion doses of 10 and 20 µg/ml provoked decreases in a- and b-wave amplitudes, which gradually decreased from 3 days following exposure and were still apparent at 28 days [25]. The a- and b- wave amplitudes decreased to approximately 40–50% of initial responses with 10 µg/mL melphalan, and to 20–30% of initial responses with 20 µg/ml melphalan. Using 5.9 µg/ml as a plausibly ideal melphalan dose, and the average human vitreous volume of 3.8 ml [26], the optimal vitreous dose of melphalan is extrapolated to be 22 µg.

Given fears of extraocular seeding of retinoblastoma with breach of the scleral surface, it has been difficult to determine vitreous levels of drugs following OAC in human patients. However, preclinical studies in non-tumor bearing pigs demonstrate that 7 mg of melphalan or 1 mg topotecan via OAC results in a vitreous Cmax of 170.1 ng/mL or 131.8 ng/mL, respectively (both concentrations exceed the IC50 determined in vitro for retinoblastoma cell lines) [1]. It is unclear how the presence of tumor or neoadjuvant focal therapy would influence these vitreous levels in humans. Munier et al. have probably come closest to correlating intravitreal melphalan dose to retinal toxicity. However confounding factors were present and ERG measurements were not obtained [27]. In that study, doses of 20–30 µg melphalan were injected into the vitreous of retinoblastoma patients every 7–10 days, up to 8 injections. It was noted that toxicity was “isolated to site of injection with salt and pepper retinopathy” in 43% of patients.

In our series, by univariate analysis, melphalan was associated with a modest change in ERG amplitude at both 3 and 12 months. A cumulative dose of 14 mg melphalan was the estimated cutpoint for change in ERG amplitude at 3 months; however these results should be interpreted with caution as there is variability in the data and only a small number of patients showed this effect. Tentative as these findings are, we want to alert our readers on the side of caution. We suggest that cumulative melphalan doses beyond this range be administered with the knowledge that there may be additional associated risk of retinal toxicity.

### Effect of Topotecan

Reports on the ocular toxicity of topotecan are mainly restricted to animal studies. Following subconjunctival injections of 10 µg in mice and periocular injections of 1 mg in rabbits, there was no toxicity on fundoscopic exam nor on histopathology [28]. Furthermore, rabbit eyes injected with four weekly doses of either 0.5 or 5 µg topotecan showed no significant change in their ERG implicit times or amplitude compared with controls injected with saline [29]. With periocular injections in human patients, the only reported local toxicities occurred in the anterior segment, including conjunctival congestion and eyelid chemosis [30]. There have been no reported ERG findings following isolated topotecan to the eye.

In our series, by univariate analysis, topotecan was associated with a minimal change in ERG amplitude at both 3 and 12 months.

### Effect of Carboplatin

Of all three drugs, the local toxicity profile of carboplatin is probably the most reported on in the literature. Periocular injections at doses of 20 mg carboplatin in retinoblastoma patients have resulted in transient periorbital edema, pseudocellulitis, fat necrosis, fibrosis, mechanical restriction, and optic atrophy [31]–[35]. Histopathological examination led one group to postulate a toxic effect on vascular endothelial cells of pial vessels [32]. In humans, toxic involvement of the choroid and retina is less well described (although it has occurred following systemic administration). However, changes demonstrated in animal studies may suggest a dose-dependent impact of carboplatin on the choroid and retina. In mice, periocular injections of 100 µg, 300 µg or .66 mg (in fibrin sealant) of carboplatin found no toxic effects evident on histopathology; and 10 mg peribulbar injections in primates had no “ill effects” on dilated examination [36]. However, 1.23 mg carboplatin in fibrin sealant injected periocularly in mice revealed severe histopathologic toxicity with necrosed retina at best, and diffusely thinned retina or no trace of it at worse [36].

This dose dependent toxicity of carboplatin is corroborated by ERG findings in animal models. In 1988, Ziolba et al. looked at ERG responses eleven days following intravitreal carboplatin and determined no change with 3 µg, a 25% decrease in scotopic repsonses with 6 µg, “minor” retinal damage by light microscopy with 10 µg and “major” retinal damage with 25 µg [37]. In a similar study the following decade, 10 µg was found to induce a negative b-wave at 7 days post injection while 100 µg had a non-progressive negative b-wave at 1 day post-injection and 1000 µg caused an extinguished response at 1 week. Histopathlogical correlation found toxicity at 10 µg and higher evidenced as “cellular damage and synaptic disruption in the outer retinal layers” – despite the ERG suggesting b-wave or bipolar cell involvement [38]. However, in 2004, work by Pardue et al. again found ERG responses suggestive of inner retinal toxicity but discovered no changes on light microscopy. In this study, subconjunctival carboplatin (12.2 µg/mL in basic salt solution or 25.1 µg/mL in fibrin sealant) in rabbits resulted in decreased b-wave amplitudes beginning at day 2 and resolving by 2 weeks [39]. While not always consistent with histopathologic correlation, the literature suggests carboplatin has a dose-dependent, inner retinal effect measurable by ERG starting at 10 µg in rabbits (equivalent of 5.88 µg/ml), which may be temporary at certain doses.

In our series, by univariate analysis, carboplatin was associated with a minimal change in ERG amplitude at both 3 and 12 months. Furthermore, by multivariate analysis, carboplatin does not appear to adversely effect the change in ERG response at either 3 or 12 months (table 4). The lack of toxicity from carboplatin may reflect the smaller sample size and insufficient data to power this analysis or the conservative doses employed in this study – the maximum dose of carboplatin was 50 mg; however, this does not exclude the possibility of toxicity at higher doses.

### Conclusion

While the data do not show a consistent fall-off in ERG responses above the cut-points, nearly all of the instances where ERG responses were substantially reduced following treatment occurred with drug exposures above the cutpoint values. While these results should be interpreted with caution, they suggest a possibly diminished degree of safety at the higher doses.

Melphalan has the strongest, and carboplatin the weakest association with decrements in ERG response. By multivariate analysis, maximum and cumulative melphalan have a modest, temporary effect on the ERG amplitude change, which is apparent at 3 months but no longer evident at 12 months after completing treatment (although the latter may be due to a small sample size). These changes are small in most cases, but would benefit from future investigations to correlate with visual acuity. By multivariate analysis, topotecan and carboplatin do not appear to adversely effect the change in ERG response. These conclusions apply over the dose ranges used here, and do not mean to imply that doses can be increased without consequence: in fact, our practice is to reduce drug dose at the first sign of toxicity to the extent consistent with satisfactory tumor response.
